# Establishment of the hematology reference intervals in a healthy population of adults in the Northwest of Morocco (Tangier-Tetouan region)

**DOI:** 10.11604/pamj.2018.29.169.13042

**Published:** 2018-03-23

**Authors:** Saad Bakrim, Youssef Motiaa, Mohamed Benajiba, Ali Ouarour, Azlarab Masrar

**Affiliations:** 1 Laboratory of Biology and Health, Abdelmalek Essaâdi University, Faculty of Science, BP 2121, Tetouan, Morocco; 2 Hematology Laboratory, Provincial Hospital Center, Mohammed VI hospital, M’diq, Morocco; 3 Hematology Laboratory, Faculty of Medicine and Pharmacy, Mohammed V University, Rabat, Morocco; Central Hematology Laboratory, Ibn Sina University Hospital, Rabat, Morocco; 4 Anesthesia Reanimation Department, Ibn Sina University Hospital, Rabat, Faculty of Medicine and Pharmacy, Mohammed V University, Rabat, Morocco; 5 National Center of Blood transfusion and Hematology, Rabat, Morocco

**Keywords:** Reference intervals, blood count, hematology, adults, Northwest Moroccan population

## Abstract

**Introduction:**

Among the most useful biological examinations in common medical practice, blood count is the most prescribed. The reference intervals of the hematological parameters of this examination are of major importance for clinical orientations and therapeutic decisions. In Morocco, the reference values used by the laboratories of medical biology and used by doctors are ones collected from Caucasian and European individuals. These values could be different in the Moroccan population. Besides, reference intervals of the blood count specific to the various Moroccan regions are missing. We decided to determine the reference intervals from a population of healthy adults of the Tangier-Tetouan region by following the procedures recommended by the IFCC-CLSI guidelines in 2008 and comparing them to those of the literature.

**Methods:**

Blood samples were taken from 15840 adult volunteers (8402 men from 18 to 55 years old and 7438 women from 18 to 50 years old) from the regional transfusion center of Tangier and Tetouan during a period between November 2014 and May 2016. The complete blood count was measured by the Sysmex KX21N^®^ analyzer. For each sample a systematic blood smear was done to determine the leukocyte differential. The data analysis was made by the software SPSS 20.0 by using percentiles 2.5^th^ and 97.5^th^.

**Results:**

A significant difference between both sexes was noted (p<0,001) for all the hematological parameters (red blood cells, hematocrit, hemoglobin, mean corpuscular volume, mean corpuscular hemoglobin, mean corpuscular hemoglobin concentration, leukocytes, neutrophils, basophils, eosinophils, monocytes, platelets and mean platelet volume) except for the numeration of lymphocytes (p = 0.552). The values of this study were compared with those reported in Arabic, Caucasian and African populations. Said comparisons showed the existence of significant differences.

**Conclusion:**

This study tries to accentuate the necessity of proceeding with the establishment of reference intervals specific to the blood count of the Moroccan population to avoid errors of diagnosis, allow clinicians to interpret with greater specificity the hematological examinations and to improve the quality of medical care distributed to patients.

## Introduction

The exploration of hematopoiesis begins routinely with the establishment of the complete blood count which provides the erythrocytes, leukocytes and platelet values of an individual as well as the morphological characteristics of these blood cells [1]. Indeed, a wide variety of pathologies can result in modifications of the blood count [2]. It is, undoubtedly, one of the most prescribed biological examinations and among the most useful in common medical practice for the evaluation of the state of health in not only sick but also healthy subjects. It is by means of this examination that specific diagnoses can be suggested and that a hematological affliction can be revealed at an early stage during clinical care. For biological analyses, this multiparameter test, prescribed by clinicians has meaning only in comparison to the results obtained from reference values which establish a major mark for clinical interpretation. The reference values are the various values of results of biological tests produced by a large population of healthy people. They appear in the form of an interval with a lower limit and superior limit determined according to the recommendations of the new concept of reference values stemming from numerous works of learned societies, in particular the International Federation of Clinical Chemistry and Laboratory Medicine (IFCC-LM) and the French Company of Clinical Biology. These recommendations aim at standardizing, harmonizing and making more rigorous the presentation of the results and improving their interpretation by clinicians [3-5].

These reference values of hematological parameters of the healthy subjects see changes according to the analytical and pre-analytical variability due to the use of different measurement systems or several factors such as age, sex, height, environment, race, pregnancy, nutritional state, ethnic origin, lifestyle, biorhythms or the consumption of tobacco, alcohol or medicine [6, 7]. For that purpose, their determination for every country, even every region, is of major importance. In Morocco, the reference values used in the laboratories of medical biology and by doctors are the ones collected from Caucasian individuals from industrialized countries with different physical, biological, environmental and behavioral characteristics. Other values are from treaties of hematology and data derived from brochures of the reagent kits which makes the reliability of the reference values questionable. If these values were looking at the Moroccan population, they could turn out very differently. Besides, the research of reference intervals of the complete blood counts specific to the various Moroccan regions would be of a certain interest because they would allow verification of the impact of geographical localization and ethnic composition. In order to respond this deficit, we suggested conducting a study with the aim of determining the reference intervals for blood count test in a population of healthy adults from the region of Tangier-Tetouan (northwest of Morocco) by following the procedures and recommendations of the IFCC-CLSI guidelines C28-A3 of 2008 [8]. The idea is to compare the values found in the present study to the values reported in the literature and those of other populations as well as to estimate the influence of age and sex on our population of study.

## Methods

The pre-analytical phases and the analytics of our study were conducted according to the international recommendations of the IFCC-LM and the CLSI of the United States (International Federation of Clinical Chemistry-Laboratory Medicine and Clinical and Laboratory Standards Institute) relative to the establishment of the reference intervals [3, 4, 8]. These recommendations stipulate that the choice of individuals in the production of reference values requires establishing precise criteria of inclusion and exclusion according to pre-analytical and biological factors of variation susceptible to interference and to decide on the number of reference individuals necessary for statistical processing of the obtained results.

**Study site**: Located only 14 kilometers from the European continent, the region of Tangier-Tetouan (geographic co-ordinates: 35°46'00" North, 5°48'00" West) was one of the sixteen regions of Morocco before the territorial cutting of 2015. It is situated at the junction of two seas in the extreme northwest of the Kingdom of Morocco, in the Rif's mountain range. It is limited, in the North by the Strait of Gibraltar and Mediterranean Sea, on the West by the Atlantic Ocean, and in the South by the region of the Gharb Chrarda Bni Hssen and in the East by Taza Al Houceima's-Taounate region. Its position on two coastal facades and the presence of reliefs of important size and average heights, the bio- geographical context of the Tangier-Tetouan region makes it Mediterranean climate moderated with oceanic influence. This region is one of the most fertile zones in Morocco with a rate of rainfall between 1000 and 1800 mm/year [9]. It extends over an area of 20 360 km^2^ with a population of 3 157 075 inhabitants according to the general census of the population and housing environment of 2015. It also records a density of 155 inhabitants /km^2^. The region consists of two prefectures (M'diq-Fnideq and Tangier-Assilah) and five provinces (Tetouan, Chefchaouen, Fahs-Anjra, Larache and Ouazzane) [10].

**Reference population**: The selection of the reference sample is the key to the success of a precise determination of reference values [11]. In total, 15840 samples corresponding to voluntary blood donors (VBD) who belong to various socio-professional groups were initially analyzed in this study. The samples came from the Regional Centers of Blood Transfusion (RCBT) of Tangier and from Tetouan as well as from mobile campaigns of the donation of blood organized in various cities of the region: Tangier, Assilah, Tetouan, Chefchaouen, M'diq, Fnideq, and Martil. Our reference sample consisted of 8402 men, 18 to 55 years old and 7438 women, 18 to 50 years old, the period of study was spread out from November, 2014 until May, 2016. The theoretically healthy adult volunteers (chosen according to the Moroccan law of the donation of blood) completed a questionnaire and benefited systematically, before every blood drive, from a medical examination with a pre-donation interrogation eliminating any suspicion of diseases or visible pathologies [12]. We excluded all situations which could affect the blood count parameters. The criteria of exclusion in our study were: the clinical criteria: hematological, hemorrhagic or thrombotic disease histories, drug administration, smoking, alcoholism, pregnant, menopausal or women using contraception, positive serological tests of viral infections; the biological criteria: morphological anomalies of the figurative elements of the blood observed in the blood smear (hypochromia, target red blood corpuscles, Plasmodium, etc). By adopting these criteria, only 14965 reference subjects were finally retained for the study among which 7930 men and 7035 women. It is necessary to note, nevertheless, that our conditions of study were unable to ensure the exclusion of possible patients presenting an iron deficiency and/or affected by thalassemia/hemoglobin diseases. Every subject participating in the study gave their consent freely according to ethical standards. This study was approved by the Regional Health Committee of the Tangier-Tetouan-Al Hoceima region.

**Blood sampling**: In our study, we followed the standard protocol of taking and preparing of blood samples to minimize the interpersonal variability. To avoid the daily variations of the blood cell parameters and those associated to physical exercise which influences the rate of hemoglobin and that of the leukocytes, we chose a morning schedule for the blood sampling (between 8 and 12 am) after a rest of at least 15 minutes [13, 14]. For every VBD, blood samples were withdrawn before the collection of blood from the antecubital vein, in system BD Vacutainer^®^ tubes (13×75 mm) of 5ml containing an anticoagulant the K3-EDTA. Analysis of the samples was performed the same day within 6 hours of collection. Immuno-hematological and serological tests were carried out on every blood donor in the RCBT laboratories of Tangier and Tetouan to identify the ABO and Rhesus blood group, syphilis serology (TPHA: Treponema Pallidum Hemagglutinations Assay), and ELISA (Enzyme Linked Immuno Sorbent Assay) detection of the Hepatitis B virus (HBV), Hepatitis C virus (HCV) and Human immunodeficiency virus (HIV).

**Hematological analysis**: A complete blood count was performed using the automate hematology analyzer Sysmex KX21N^®^ (Sysmex Corporation, Kobe, Japan) at the laboratory of hematology of the hospital Mohamed VI of M'diq. The KX-21N^®^ is constituted by three detectors: the number of white blood cells (WBC) is determined by the detector GB using the method of detection CC, the number of red blood cells (RBC) and platelets (PLT) is produced by the detector GR also using the method of detection CC and the detector HB measures the concentration of hemoglobin by using the method of non-cyanide hemoglobin. The KX-21N^®^ handles approximately 60 samples per hour and allows to specify 19 blood parameters: WBC, RBC, PLT, lymphocytes (LYM), neutrophils (NEU), hemoglobin (HGB), hematocrit (HCT), mean corpuscular volume (MCV), mean corpuscular hemoglobin (MCH), mean corpuscular hemoglobin concentration (MCHC), index of distribution of the RBC by standard deviation (IDR-SD), index of distribution of the RBC by coefficient of variation (IDR-CV), index of distribution of platelets (IDP), mean platelet volume (MPV), platelet index (P-LCR) and the percentage of the LYM, the NEU and that of the mixed cells (MXD) consisted of monocytes (MON), basophils (BAS) and eosinophils (EOS) [15].

For every series of analyses, the validity of the measures was guaranteed by the passage of three sample witnesses: a normal blood witness, a low blood pathological witness and a third higher one. The results obtained by these samples have to become integrated with values supplied by the manufacturer for samples witnesses. For every VBD, a blood smear was produced by spreading a small drop of blood freshly taken from an EDTA tube, and colored by the May-Grünwald-Giemsa (MGG). The parameters studied in the optical microscope were: 1) The RBC morphology to detect possible corpuscular anomalies. 2) The leukocyte parameter was determined, double-blind, by two different operators. Each of them established the percentage of the various leukocyte population (NEU, EOS, BAS, MON and LYM) on 200 leukocyte elements. In case of a difference of more than 5 cells for a leukocyte population, formulas were double-checked by two other readings (the same operators). The final formula was established by taking the average of both formulas. The values absolved from the NEU, EOS, BAS, LYM and MON, expressed in 10^9^/L, were deducted from the leukocyte numeration measured by the automate. 3) Platelets studied to research morphological anomalies or platelet aggregates.

**Statistical analysis**: The data were analyzed by means of the software SPSS 20.0 (Inc, Chicago, Him). The studied group was distributed according to sex and age: 18-29 years, 30-39 years and 40-50 years for the women and 18-29 years, 30-39 years, 40-50 years and 51-55 years for the men. The study of the distribution of variables was made by the test of Kolmogorov-Smirnov. The quantitative variables were expressed by the median, standard deviation and percentiles 2.5^th^ and 97.5^th^, and were used to limit the reference intervals. The qualitative variables were expressed in numbers and percentage. The comparison of the quantitative variables was made using the test of Mann-Whitney for two groups and the test of Kruskall-Wallis for more than two groups complemented by a correction by the test of Benferroni when the difference is significant. The comparison of the qualitative variables was made using the test of Khi-2. A difference is considered as statistically significant if p < 0.05.

## Results

**Characteristics of the reference population**: From a total of 15840 VBD, our reference population consisted of 14965 Moroccan VBD distributed in 7930 men from 18 to 55 years old and 7035 women from 18 to 50 years old with a sex-ratio (M/F) equal to 1:13. Samples excluded from the study were for the following reasons: 1) Positive serology (HBV) (HCV) (TPHA) (HIV): 150 women and 324 men. 2) Globular anomalies of erythrocyte on smears:-148 men: hypochromia (39 subjects), anisocytosis (76 subjects) and target RBC (33 subjects). - 253 women: hypochromia (54 subjects), anisocytosis (154 subjects) and target RBC (45 subjects). The distribution of the VBD according to sex, provinces and age are represented in Table 1. The mean age of male VBD was 31.75 ± 10.67 years while that of female was 28.86 ± 8.26 years.

**Table 1:** Distribution of the studied population according to sex, provinces and age

		District									
Sex	Age (Years)	Tangier-Assilah		Tetouan		M'diq-Fnideq		Chefchaouen		Overall	
		n	%	n	%	n	%	n	%	n	%
**Males**	18-29	1735	52	1036	53.3	618	46.9	631	47.3	4020	50.7
	30-39	861	25.8	438	22.6	297	22.5	330	24.7	1926	24.3
	40-50	405	12.1	280	14.4	247	18.7	217	16.3	1149	14.5
	51-55	333	10	188	9.7	157	11.9	157	11.8	835	10.5
	Total	3334	100	1942	100	1319	100	1335	100	7930	100
**Females**	18-29	1574	57.5	1053	55.7	738	55.5	696	64.8	4061	57.7
	30-39	683	24.9	539	28.5	385	28.9	211	19.6	1818	25.8
	40-50	482	17.6	300	15.9	207	15.6	167	15.5	1156	16.4
	Total	2739	100	1892	100	1330	100	1074	100	7035	100

**Blood count results according to sex**: The means, standard deviations, medians and the reference intervals of the various parameters of the blood count test according to sex are presented in the Table 2. A significant difference between both sexes was noted (p<0,001) with regard to all the studied hematological parameters: RBC, HGB, HCT, MCV, MCH, MCHC, WBC, MON, NEU, EOS, BAS, PLT and MPV except the LYM (p = 0.552). We noted that the values of the median erythrocyte parameters for men were higher than those for women, RBC 5.1×10^12^/L (4.37-5.96×10^12^/L) for men versus 4.5×10^12^/L (3.86-5.20×10^12^/L) for women, HGB 15.0 g/dL (13-17.1 g/dL) for men versus 13.0 g/dL (11-14.8 g/dL) for women, HCT 44.1% (38.3-50%) for men versus 38.6% (33.5-43.9%) for women, MCV 86.5 fL (77.4-94.2 fL) for men versus 86.2 fL (75.1-94.7 fL) for women, MCH 29.6 pg (25.2-32.3 pg) for men versus 29.2 pg (24-32.3 pg) for women and MCHC 34.1 g/dL (31.7-36 g/dL) for men versus 33.7 g/dL (31.2-36 g/dL) for women. The leukocyte parameter values were expressed in absolute values (#) and not in percentages (%). The median value of the leukocytes was 6.9×10^9^/L (4.1-10.8×10^9^/L) for men and 7.0×10^9^/L (4.1-10.7×10^9^/L) for women (p < 0.001). For NEU, the median value was 3.8×10^9^/L (1.8-7.0×10^9^/L) for men and 3.9×10^9^/L (1.8-7.0×10^9^/L) for women (p<0.001). For EOS, the median value was 00×10^9^/L (0.0-0.6×10^9^/L) for men and 0.1×10^9^/L (0.0-0.5× 10^9^/L) for women (p<0.001). For BAS and LYM, the values of the median were identical for man and for woman, 00×10^9^/L (0.00-0.08×10^9^/L) (p<0.001) and 2.2×10^9^/L (1.2-3.8 × 10^9^/L) (p = 0.552), respectively. For MON, the median value was 0.6×10^9^/L (0.2-1.2×10^9^/L) for men and 0.5×10^9^/L (0.2-1.2×10^9^/L) for women (p < 0.001). For the platelet numeration, the values of the median for women were slightly superior to those observed for men. They were 218×10^9^/L (145-338×10^9^/L) for men and 237× 10^9^/L (150-378×10^9^/L) for women (p < 0.001). The value of the median MPV was 11.5 fL (9.4-13.7 fL) for men and 11.2 fL (9-13.7 fL) for women (p < 0.001).

**Table 2:** Means, standard deviations, medians and reference intervals of the various parameters of the complete blood count of the studied population according to sex

Hematological parameters	Sex								*P value*
	Females				Males				
			Reference interval				Reference interval		
	Mean (SD)	Median	Percentile 2.5	Percentile 97.5	Mean (SD)	Median	Percentile 2.5	Percentile 97.5	
RBCx10^12^/L	4.51 (0.35)	4.5	3.86	5.2	5.12 (0.4)	5.1	4.37	5.96	<0.001 ^a^
HGB (g/dL)	13.01 (0.94)	13	11	14.8	15.01 (1.05)	15	13	17.1	<0.001 ^a^
HCT (%)	38.61 (2.72)	38.6	33.5	43.9	44.07 (3.04)	44.1	38.3	50	<0.001 ^a^
MCV (fL)	85.82 (4.92)	86.2	75.1	94.7	86.26 (4.36)	86.5	77.4	94.2	<0.001 ^a^
MCH (pg)	28.93 (2.07)	29.2	24	32.3	29.4 (1.84)	29.6	25.2	32.3	<0.001 ^a^
MCHC (g/dL)	33.69 (1.22)	33.7	31.2	36	34.06 (1.12)	34.1	31.7	36	<0.001 ^a^
WBCx10^9^ /L	7.12 (1.68)	7	4.1	10.7	7.04 (1.72)	6.9	4.1	10.8	<0.001 ^a^
LYMx10^9^/L	2.33 (0.65)	2.2	1.2	3.8	2.32 (0.66)	2.2	1.2	3.8	0.552 ^b^
MONx10^9^ /L	0.59 (0.3)	0.5	0.2	1.2	0.66 (0.31)	0.6	0.2	1.2	<0.001 ^a^
NEUx10^9^/L	4.08 (1.35)	3.9	1.8	7	3.96 (1.33)	3.8	1.8	7	<0.001 ^a^
EOSx10^9^/L	0.13 (0.17)	0.1	0	0.5	0.12 (0.17)	0	0	0.6	<0.001 ^a^
BASx10^9^/L	0.008(0.03)	0	0	0.08	0.006 (0.02)	0	0	0.08	<0.001 ^a^
PLTx10^9^/L	243.5 (58.4)	237	150	378	223.05 (51.3)	218	145	338	<0.001 ^a^
MPV (fL)	11.2 (1.19)	11.2	9	13.7	11.49 (1.10)	11.5	9.4	13.7	<0.001 ^a^

SD, standard deviation; RBC, red blood cell; HGB, hemoglobin; HCT, hematocrit; MCV, mean corpuscular volume; MCH, mean corpuscular hemoglobin; MCHC, mean corpuscular hemoglobin concentration; WBC, white blood cell; LYM, lymphocytes; MON, monocytes; NEU, neutrophils; EOS, eosinophils; BAS, basophils; PLT, platelets; MPV, mean platelet volume.

Mann-Whitney U-test for nonnormally distributed parameters was done between males and females: All hematological parameters except lymphocytes showed significant differences between females and males.

*p* < 0.05 was considered as statistically significant.

a statistically significant.

b statistically non-significant.

**Blood count results according to age**: The means, standard deviations, medians and reference intervals of the various parameters of the blood count for men and women according to age groups are presented, respectively, in Table 3 and Table 4. The study showed variations of certain erythrocyte parameters according to age. A significant difference of the values of HGB, HCT, MCV, MCH, MCHC according to age was noted (p < 0.001) for men except for the RBC (p = 0.062). In the women's group, the HGB and the HCT showed a significant difference according to age (p = 0.002 and p<0.001, respectively). For other erythrocyte parameters, we noticed no significant difference, according to age. For leukocyte lineage, the study revealed a slight decrease in the total number of the WBC for the age between 51-55 years for men and the age group 40-50 years for women, but this decrease was not statistically significant for either sexes. The value of the median number of the NEU in each of the age groups was higher for woman than for men without any of significant difference according to age for both sexes. We observed a significant difference according to age of men in the rates of LYM, MON and EOS (p = 0.009, p = 0.002 and p = 0.003, respectively) while there was no significant difference in the number of BAS with age (p = 0.093). On the other hand, in the female genital organ, we noticed a significant difference according to age of women in the number of MON (p = 0.028) and BAS (p < 0.001) while that of the LYM and the EOS were not statistically significant. The values of the median platelet numeration according to age were superior in women with regard to men. According to age, this difference was statistically significant for men (p = 0.001) and for women (p = 0.002). On the other hand, the values of the median MPV according to age were superior for men than for women. This difference according to age was not statistically significant for men (p = 0.699) nor women (p = 0.257).

**Table 3:** Values of the complete blood count parameters for men according to age

	Males												*P value*
Age groups	18-29 years old (n=4020)			30-39 years old (n=1926)			40-50 years old (n=1149)			51-55 years old (n=835)			
Hematologicalparameters			Reference interval			Reference interval			Reference interval			Reference interval	
	Mean (SD)	Median	Percentile 2.5-97.5	Mean (SD)	Median	Percentile 2.5-97.5	Mean (SD)	Median	Percentile 2.5-97.5	Mean (SD)	Median	Percentile 2.5-97.5	
RBCx10^12^/L	5.11 (0.39)	5.11	4.36-5.93	5.13 (0.40)	5.11	4.39-5.99	5.14 (0.40)	5.11	4.41-6.02	5.09 (0.41)	5.07	4.36-5.99	0.062^b^
HGB (g/dL)	15.00 (1.07)	15	13-17.1	15.07 (1.03)	15.1	13-17.2	15.09 (0.98)	15	13.2-17	1482 (1.01)	14.8	13-17	<0.001^a^
HCT (%)	43.98 (3.09)	44	38.2-50.2	44.17 (2.91)	44.2	38.4-50.1	44.39 (3.17)	44.3	39.1-49.9	43.81 (2.87)	43.9	38.3-49.5	<0.001^a^
MCV (fL)	86.16 (4.29)	86.4	77.4-94.1	86.32 (4.35)	86.6	77.5-93.9	86.56 (4.40)	86.8	77.2-94.6	86.22 (4.60)	86.4	76.7-94.4	0.004 ^a^
MCH (pg)	29.39 (1.82)	29.6	25.5-32.4	29.46 (1.86)	29.7	25.2-32.3	29.46 (1.80)	29.7	25.3-32.3	29.17 (1.95)	29.4	24.6-32.3	<0.001^a^
MCHC (g/dL)	34.10 (1.13)	34.1	31.8-36.1	34.10 (1.11)	34.2	31.7-36.1	34.03 (1.10)	34.1	31.8-35.9	33.82 (1.07)	33.9	31.5-35.9	<0.001^a^
WBCx10^9^ /L	7.04 (1.70)	6.9	4.1-10.8	7.07 (1.76)	6.9	4-11.1	7.04 (1.70)	6.9	4.1-10.8	6.98 (1.74)	6.8	4.1-10.9	0.710 ^b^
LYMx10^9^/L	2.32 (0.65)	2.3	1.3-3.8	2.34 (0.66)	2.3	1.2-3.8	2.32 (0.65)	2.2	1.2-3.8	2.27 (0.70)	2.2	1.2-3.9	0.009 ^a^
MONx10^9^ /L	0.65 (0.31)	0.6	0.2-1.2	0.67 (0.30)	0.6	0.2-1.2	0.68 (0.31)	0.7	0.2-1.2	0.67 (0.30)	0.7	0.2-1.2	0.002 ^a^
NEUx10^9^/L	3.97 (1.32)	3.8	1.8-6.9	3.96 (1.35)	3.8	1.7-7.1	3.93 (1.31)	3.8	1.7-7	3.95 (1.33)	3.8	1.8-7.1	0.821 ^b^
EOSx10^9^/L	0.13 (0.18)	0	0-0.6	0.12 (0.17)	0	0-0.5	0.11 (0.17)	0	0-0.6	0.12 (0.18)	0	0-0.6	0.003 ^a^
BASx10^9^/L	0.01 (0.02)	0	0-0.08	0.00 (0.02)	0	0-0.07	0.01 (0.02)	0	0-0.08	0.01 (0.02)	0	0-0.08	0.093 ^b^
PLTx10^9^/L	223.12 (51.5)	218	146-340	221.97 (50.8)	217	144-333	219.89 (49.1)	215	145-334	229.56 (53.67)	224	146-349	0.001 ^a^
MPV (fL)	11.50 (1.10)	11.5	9.4-13.7	11.47 (1.12)	11.5	9.4-13.7	11.48 (1.09)	11.4	9.4-13.8	11.45 (1.07)	11.4	9.5-13.6	0.699 ^b^

SD, standard deviation; RBC, red blood cell; HGB, hemoglobin; HCT, hematocrit; MCV, mean corpuscular volume; MCH, mean corpuscular hemoglobin; MCHC, mean corpuscular hemoglobin concentration; WBC, white blood cell; LYM, lymphocytes; MON, monocytes; NEU, neutrophils; EOS, eosinophils; BAS, basophils; PLT, platelets; MPV, mean platelet volume.

Statistical comparison: Chi squared test and Kruskal-Wallis test for nonnormally distributed parameters was done between the four age groups with the Bonferroni method adjustment of *p* value.

*p< 0.05* was considered as statistically significant.

a statistically significant.

b statistically non-significant

**Table 4:** Values of the complete blood count parameters for women according to age

	Females												*P value*
Age groups	18-29 years old (n=2638)				30-39 years old (n=2342)				40-50 years old (n=2055)				
Hematological parameters				Reference interval				Reference interval				Reference interval	
	Mean	SD	Median	Percentile 2.5-97.5	Mean	SD	Median	Percentile 2.5-97.5	Mean	SD	Median	Percentile 2.5-97.5	
**RBCx10^12^/L**	4.51	0.34	4.5	3.88-5.19	4.50	0.36	4.49	3.82-5.19	4.52	0.35	4.51	3.87-5.22	0.088 ^b^
**HGB (g/dL)**	13.01	0.91	13	11.1-14.7	12.96	0.98	13	10.9-14.8	13.06	0.91	13	11-14.8	0.002 ^a^
**HCT (%)**	38.60	2.66	38.5	33.7-43.9	38.47	2.77	38.4	33.3-44.1	38.80	2.71	38.8	33.6-44	<0.001 ^a^
**MCV (fL)**	85.77	4.77	86.15	75.3-94.3	85.73	5.09	86.1	74.6-94.6	85.98	4.92	86.2	75.2-95.4	0.296 ^b^
**MCH (pg)**	28.92	1.99	29.2	24.5-32.2	28.90	2.19	29.2	23.6-32.4	28.96	2.04	29.2	24.0-32.3	0.495 ^b^
**MCHC (g/dL)**	33.69	1.19	33.7	31.3-36	33.69	1.27	33.8	31-36	33.68	1.21	33.7	31.3-36	0.633 ^b^
**WBCx10^9^ /L**	7.14	1.70	7	4.1-10.7	7.15	1.70	7	4.1-10.8	7.05	1.62	6.9	4.1-10.5	0.171 ^b^
**LYMx10^9^/L**	2.33	0.65	2.2	1.3-3.8	2.34	0.67	2.3	1.2-3.8	2.30	0.64	2.2	1.24-3.8	0.094 ^b^
**MONx10^9^ /L**	0.58	0.30	0.5	0.2-1.2	0.59	0.30	0.6	0.2-1.2	0.60	0.30	0.6	0.2-1.2	0.028 ^a^
**NEUx10^9^/L**	4.10	1.39	4	1.8-7.1	4.10	1.36	3.9	1.9-7.1	4.02	1.30	3.9	1.8-6.9	0.117 ^b^
**EOSx10^9^/L**	0.13	0.16	0.05	0-0.5	0.14	0.17	0.1	0-0.5	0.14	0.18	0.1	0-0.6	0.054 ^b^
**BASx10^9^/L**	0.01	0.03	0	0-0.09	0.01	0.02	0	0-0.08	0.01	0.02	0	0-0.08	<0.001 ^a^
**PLTx10^9^/L**	246.2	59.4	241	150-380	240.61	57.27	233	150-373	243.29	58.75	237	149-378	0.002 ^a^
**MPV (fL)**	11.19	1.17	11.2	9-13.6	11.19	1.22	11.2	8.9-13.7	11.23	1.18	11.2	9-13.7	0.257 ^b^

SD, standard deviation; RBC, red blood cell; HGB, hemoglobin; HCT, hematocrit; MCV, mean corpuscular volume; MCH, mean corpuscular hemoglobin; MCHC, mean corpuscular hemoglobin concentration; WBC, white blood cell; LYM, lymphocytes; MON, monocytes; NEU, neutrophils; EOS, eosinophils; BAS, basophils; PLT, platelets; MPV, mean platelet volume.

Statistical comparison: Chi squared test and Kruskal-Wallis test for nonnormally distributed parameters was done between the four age groups with the Bonferroni method adjustment of *p* value.

*p< 0.05* was considered as statistically significant.

a statistically significant.

b statistically non significant

**Blood count results according to the studied provinces**: The means, standard deviations, medians and reference intervals of various blood count parameters for men and women according to the studied provinces are presented in Table 5 and Table 6, respectively. We noted a variation of the erythrocyte parameters according to the four studied provinces. Median values of RBC, HGB, HCT, MCV, MCH and MCHC were superior in men compared to results of women. A significant difference, according to provinces was observed for all the erythrocyte parameters RBC, HGB, HCT, MCV, MCH, MCHC for women (p < 0.001) while in the male population the differences of MCV (p = 0.949), MCH (p = 0.428) and MCHC (p = 0.244 ) were not statistically significant. Concerning leukocyte lineage, in males we observed a significant difference between the studied provinces in rates of WBC, LYM, MON, EOS and BAS (p = 0.020, p < 0.001, p = 0.002, p < 0.001 and p < 0.001, respectively) while no significant difference of the number of NEU was observed (p = 0.486). For women, we noted a significant difference between the four provinces in the number of WBC and MON (p = 0.005) and the LYM and BAS (p < 0.001) while that of NEU (p = 0.136) and EOS (p = 0.418) was not statistically significant. The median values of the platelet numeration according to provinces were superior for women than for men. This difference according to provinces was statistically significant for women (p = 0.045) and not significant for men (p = 0.492). The median values of the MPV according to provinces were superior for men than for women. This difference was statistically significant for men (p < 0.001) as well as for women (p = 0.006).

**Table 5:** Values of the complete blood count parameters for men according to provinces

Males													*P value*
Disctrict	Tangier-Tetouan (n=3334)			Tetouan (n=1942)			Mdiq-Fnideq (n=1319)			Chefchaouen (n=1335)			
Hematological parameters			Reference interval			Reference interval			Reference interval			Reference interval	
	Mean (SD)	Median	2.5-97.5 Percentile	Mean (SD)	Median	2.5-97.5 Percentile	Mean (SD)	Median	2.5-97.5 Percentile	Mean (SD)	Median	2.5-97.5 Percentile	
**RBCx10^12^/L**	5.13 (0.39)	5.11	4.39-5.95	5.10 (0.40)	5.09	4.33-5.92	5.08 (0.37)	5.07	4.37-5.84	5.15 (0.42)	5.12	4.4-6.1	<0.001 ^a^
**HGB (g/dL)**	15.02 (1.05)	15	12.9-17	14.97 (1.06)	15	13-17.1	14.93 (0.99)	14.9	13.1-16.9	15.13 (1.06)	15.1	13.2-17.5	<0.001 ^a^
**HCT (%)**	44.13 (2.93)	44.2	38.5-50	43.89 (3.20)	44	38.1-49.5	43.81 (2.86)	43.8	38.2-49.4	44.43 (3.20)	44.2	38.6-51	<0.001 ^a^
**MCV (fL)**	86.20 (4.52)	86.5	77.0-94.4	86.27 (4.44)	86.5	77.5-94.4	86.34 (3.87)	86.4	78.1-93.6	86.34 (4.27)	86.5	77.3-94.2	0.949 ^b^
**MCH (pg)**	29.34 (1.90)	29.6	24.9-32.3	29.45 (1.87)	29.6	25.3-32.5	29.41 (1.65)	29.6	26-32.3	29.45 (1.84)	29.6	25.3-32.3	0.428 ^b^
**MCHC (g/dL)**	34.02 (1.14)	34.1	31.5-36	34.11 (1.08)	34.2	32-36.2	34.08 (1.09)	34.1	31.9-36.1	34.06 (1.13)	34.1	31.7-36	0.244 ^b^
**WBCx10^9^ /L**	6.98 (1.68)	6.8	4.1-10.8	7.11 (1.76)	7	3.9-11	7.10 (1.69)	7	4.2-10.7	7.04 (1.78)	6.8	4.1-11	0.020 ^a^
**LYMx10^9^/L**	2.28 (0.64)	2.2	1.2-3.7	2.36 (0.67)	2.3	1.2-3.9	2.34 (0.67)	2.2	1.3-3.8	2.35 (0.67)	2.3	1.3-3.8	<0.001 ^a^
**MONx10^9^ /L**	0.65 (0.30)	0.6	0.2-1.2	0.66 (0.31)	0.6	0.2-1.2	0.67 (0.31)	0.7	0.2-1.2	0.68 (0.31)	0.7	0.2-1.2	0.002 ^a^
**NEUx10^9^/L**	3.94 (1.31)	3.8	1.8-7	3.97 (1.34)	3.8	1.7-6.9	3.99 (1.31)	3.8	1,8-7	3.93 (1.37)	3.8	1.7-6.96	0.486 ^b^
**EOSx10^9^/L**	0.12 (0.18)	0	0-0.6	0.13 (0.16)	0.09	0-0.5	0.11 (0.17)	0	0-0.6	0.12 (0.18)	0	0-0.6	<0.001 ^a^
**BASx10^9^/L**	0.007 (0.02)	0	0-0.08	0.005 (0.02)	0	0-0.08	0.004 (0.02)	0	0-0.08	0.006 (0.02)	0	0-0.08	<0.001 ^a^
**PLTx10^9^/L**	223.62 (51.74)	218	145-339	223.96 (52.5)	218	145-351	222.78 (50.04)	218	147-337	220.58 (49.73)	217	146-331	0.492 ^b^
**MPV (fL)**	11.47 (1.09)	11.4	9.4-13.7	11.46 (1.11)	11.4	9.4-13.7	11.44 (1.13)	11.4	9.3-13.7	11.60 (1.09)	11.6	9.5-13.8	<0.001 ^a^

SD, standard deviation; RBC, red blood cell; HGB, hemoglobin; HCT, hematocrit; MCV, mean corpuscular volume; MCH, mean corpuscular hemoglobin; MCHC, mean corpuscular hemoglobin concentration; WBC, white blood cell; LYM, lymphocytes; MON, monocytes; NEU, neutrophils; EOS, eosinophils; BAS, basophils; PLT, platelets; MPV, mean platelet volume.

Statistical comparison: Chi squared test and Kruskal-Wallis test for nonnormally distributed parameters was done between the four age groups with the Bonferroni method adjustment of *p* value. *p*<0.05 was considered as statistically significant.

a statistically significant.

b statistically non-significant.

**Table 6:** Values of the complete blood count parameters for women according to provinces.

Females													*P value*
Disctrict	Tangier-Tetouan (n=2739)			Tetouan (n=1892)			M'diq-Fnideq (n=1330)			Chefchaouen (n=1074)			
Hematological parameters			Reference interval			Reference interval			Reference interval			Reference interval	
	Mean (SD)	Median	2.5-95.5 Percentile	Mean (SD)	Median	2.5-95.5 Percentile	Mean (SD)	Median	2,5-95,5 Percentile	Mean (SD)	Median	2.5-95.5 Percentile	
**RBCx10^12^/L**	4.52 (0.34)	4.52	3.87-5.23	4.47 (0.34)	4.46	3.85-5.18	4.46 (0.32)	4.46	3.85-5.11	4.57 (0.36)	4.56	3.88-5.36	<0.001^a^
**HGB (g/dL)**	12.94 (0.97)	13	10.8-14.7	12.99 (0.93)	13	11-14.8	12.97 (0.85)	12.9	11.3-14.7	13.26 (0.88)	13.2	11.7-15	<0.001^a^
**HCT (%)**	38.52 (2.73)	38.5	33.3-43.9	38.46 (2.70)	38.4	33.53-43.8	38.39 (2.59)	38.3	33.7-43.3	39.4 (2.70)	39.3	34.49-45.11	<0.001^a^
**MCV (fL)**	85.3 (5.18)	85.8	73.9-94.5	86.09 (4.69)	86.3	76.33-94.8	86.09 (4.55)	86.5	76.5-94.1	86.32 (4.93)	86.6	76.59-96	<0.001^a^
**MCH (pg)**	28.68 (2.21)	29	23.4-32.3	29.09 (2.01)	29.3	24.53-32.5	29.1 (1.90)	29.3	24.6-32.27	29.05 (1.93)	29.3	24.7-32.41	<0.001^a^
**MCHC (g/dL)**	33.59 (1.27)	33.6	30.95-36	33.78 (1.18)	33.8	31.4-36.1	33.78 (1.18)	33.7	31.5-36.2	33.67 (1.17)	33.7	31.3-36	<0.001^a^
**WBCx10^9^ /L**	7.053 (1.67)	6.9	4-10.6	7.17 (1.67)	7	4.1-10.8	7.19 (1.64)	7	4.1-10.5	7.08 (1.72)	6.9	4.1-10.9	0.005 ^a^
**LYMx10^9^/L**	2.283 (0.63)	2.2	1.2-3.8	2.37 (0.66)	2.3	1.3-3.9	2.34 (0.67)	2.3	1.2-3.9	2.32 (0.65)	2.2	1.2-3.8	<0.001^a^
**MONx10^9^ /L**	0.572 (0.29)	0.5	0.2-1.2	0.59 (0.3)	0.5	0.2-1.2	0.60 (0.29)	0.6	0.2-1.2	0.60 (0.31)	0.6	0.2-1.2	0.005 ^a^
**NEUx10^9^/L**	4.075 (1.35)	3.9	1,9-7	4.08 (1.33)	3.9	1.9-7.1	4.11 (1.35)	4	1.8-7	4.01 (1.36)	3.9	1.8-7.2	0.136 ^b^
**EOSx10^9^/L**	0.135 (0.17)	0.1	0-0,5	0.13 (0.16)	0.1	0-0.5	0.12 (0.16)	0.05	0-0.5	0.13 (0.16)	0.1	0-0.6	0.418 ^b^
**BASx10^9^/L**	0.01 (0.02)	0	0-0,09	0.006 (0.03)	0	0-0.08	0.007 (0.02)	0	0-0.08	0.009 (0.02)	0	0-0.08	<0.001^a^
**PLTx10^9^/L**	245.8 (58.91)	239	150-380	242 (57.88)	235.5	150-375	241.1 (58.8)	232.5	149-379	243.1 (57.3)	239.5	149-375	0.045 ^a^
**MPV (fL)**	11.22 (1.17)	11.2	9-13.6	11.2 (1.2)	11.2	8.9-13.7	11.1 (1.21)	11.1	8.9-13.5	11.27 (1.19)	11.2	9.1-13.7	0.006 ^a^

SD, standard deviation; RBC, red blood cell; HGB, hemoglobin; HCT, hematocrit; MCV, mean corpuscular volume; MCH, mean corpuscular hemoglobin; MCHC, mean corpuscular hemoglobin concentration; WBC, white blood cell; LYM, lymphocytes; MON, monocytes; NEU, neutrophils; EOS, eosinophils; BAS, basophils; PLT, platelets; MPV, mean platelet volume.

Statistical comparison: Chi squared test and Kruskal-Wallis test for nonnormally distributed parameters was done between the four age groups with the Bonferroni method adjustment of *p* value. *p< 0.05* was considered as statistically significant.

a statistically significant.

b statistically non significant.

## Discussion

The blood count is the laboratory test most frequently prescribed in common medical practice. It offers precious information because it is able to distinguish a normal situation from a pathological one through a multitude of conditions [16]. In the Tangier-Tetouan region (northwest of Morocco), the reference intervals of the complete blood count test for healthy adult were never established. The values used currently in the region by the laboratories of medical biology and by doctors are the ones adopted from books of hematology or pulled from studies on populations of America or Western Europe [17]. This study was conducted to establish the reference intervals of various blood count parameters in a Moroccan population of healthy adults of the Tangier-Tetouan region and compare them with the normal values published in the literature as well as those relative to other countries and other regions of Morocco (Table 7). The staff of the population selected for this study counted 14965 subjects. According to the international recommendations of the IFCC-LM and the CLSI, this size of sample can be considered to be representative of the adult population of the studied provinces (number of individuals ≥ 120 for each group) [8]. The reference intervals of the blood count parameters which we looked for in this work were closer to the reference intervals determined from Caucasian [16] and Arabic populations [18]. Nevertheless, they were low compared to the limits of the references published in the literature [19]. Our intervals however, diverge in their majority comparison to intervals published in a study carried out on a Moroccan population of the region of Casablanca (Morocco) [20]. The results of the studies done on Caucasian populations are very close, although the studied populations and the techniques of measurement are different [5, 21]. The comparison of our results with those of these studies, as well for men and women, shows that in our population the reference limits are lower. The reference values of the HGB defined by Swaanenburg for a population of more than 15 years were 14.00 - 17.87 g/dL for men versus 12.60-16.40 g/dL for women [21].

**Table 7:** Comparison of reference intervals of our study with those found in the literature and those of other populations of women and men.

Hematologicalparameters	Sex : Females						Sex : Males					
	Reference interval						Reference interval					
	French (2014) (16)	Togo (2011) (25)	Textbook References (2010) (19)	Morocco Casablanca (2014) (20)	Tunisia (2012) (18)	Our study (2016)	French (2014) (16)	Togo (2011) (25)	Textbook References (2010) (19)	Morocco Casablanca (2014) (20)	Tunisia (2012) (18)	Our study (2016)
**RBCx10^12^/L**	3.96-5.12	3.1-6.0	4.0-5,4	3.6-5.6	3.66-4.90	3.86-5.2	4.39-5.68	3.3-6.4	4.5-6.0	4.1-6.0	4.22-5.59	4.37-5.96
**HGB (g/dL)**	11.70-15.00	10.3-17.1	12.0-16,0	10.4-16.8	11.06-13.87	11-14.8	13.40-16.70	10.0-18.4	13.0-18.0	12.3-17.2	12.64-15.87	13-171
**HCT (%)**	34.70-44.40	28-47	37-47	32.1-47.8	33.45-42.18	33.5-43.9	39.20-48.60	28-54	40-54	37.3-49.5	38.40-48.02	38.3-50
**MCV (fL)**	78.40-95.30	80-95	85-98	70.1-100.0	80.12-94.65	75.1-94.7	80.20-95	80-99	85-98	77.4-94.9	80.72-95.68	77.4-94.2
**MCH (pg)**	26.10-32.50	25-37	27-32	22.5-36.3	26.67-30.81	24-32.3	27.20-32.80	26-36	27-32	26.3-33.6	26.70-31.65	25.2-32.3
**MCHC (g/dL)**	31.90-35.80	30-41	32-36	31.3-38.5	31.43-34.20	31.2-36	32.40-36.30	29-39	32-36	32.4-37.0	31.70-34.25	31.7-36
**WBCx10^9^ /L**	3.91-10.88	2.2-7.8	4.0-10,0	3.3-11.9	3.92-9.57	4.1-10.7	4.08-10.81	1.9-10.1	4.0-10.0	3.5-10.2	3.48-9.50	4.1-10.8
**LYMx10^9^/L**	1.26-3.64	1.2-4.3	1.0-4,0	0.8-4.4	0.99-3.32	1.2-3.8	1.27-3.77	1.1-4.3	1.0-4.0	0.8-4.4	0.89-3.35	1.2-3.8
**MONx10^9^ /L**	0.20-0.66	0.05-0.8	0.1-1,0	0.2-1.1	0.03-0.53	0.2-1.2	0.23-0.74	0.05-0.8	0.1-1.0	0.2-1.1	0-0.59	0.2-1.2
**NEUx10^9^/L**	1.74-7.10	0.5-4.4	1.5-7,0	1.2-8.6	2.26-6.03	1.8-7	1.82-6.81	0.5-5.4	1.5-7.0	1.5-6.6	1.99-5.87	1.8-7
**EOSx10^9^/L**	0.04-0.52	0-0.5	<0.5	0.01-1.09	0-0.35	0-0.5	0.04-0.56	0-0.5	<0.5	0.0-1.0	0-0.28	0-0.6
**BASx10^9^/L**	0-0.08	NA	<0.05	0.0-0,08	0-0.09	0-0.08	0-0.09	NA	<0.05	0.0-0.08	0-0.07	0-0.08
**PLTx10^9^/L**	186-440	150-436	150-500	155-355	138.17-372.53	150-378	171-397	120-443	150-500	130-368	126.79-339.19	145-338
**MPV (fL)**	7.50-10.9	NA	NA	8.9-14.0	NA	9-13.7	7.4-10.8	NA	NA	8.9-13.1	NA	9.4-13.7

NA: Not available

In our study the reference values of the HGB were 13.00-17.1 g/dL for men versus 11.00-14.8 g/dL for women. Few studies in the literature have thus far studied Arabic populations. In a Tunisian study, Ben Amor et al. provided the reference values of the blood count test from a population of 1000 healthy VBD, between 18 to 61 years old from the region of Sfax and selected according to the rules of Tunisian blood donation [18]. The measures were made by means of Coulter^®^ ACT10 automate. At the term of their study, Ben Amor et al. published reference ranges close to intervals of standard reference determined from Caucasian populations and widely used in clinical practice. The comparison between the Tunisian study and our study of reference values concerning the number of RBC, rate of the HGB and the HCT as well as of the leukocytosis for women showed homogeneity. However, the lower limit of the values from platelet numeration was low in Tunisians (126×10^9^/L for men versus 138 × 10^9^/L for women) congruent with our study (145x10^9^/L for men versus 150x10^9^/L for women). The most important ethnic variations were observed with subjects of the black race of African or American origin [22-24]. For example, Kueviakoe et al established the reference values of a Togolese population in a study grouping 1349 HIV negative VBD. The analyses of this study were made with type Sysmex SF 3000's device^®^ [25]. Although Morocco is an African country, no homogeneity is perceptible between the values reported in the Togolese study and those of our study. Indeed, Kueviakoe et al reported lower values of the lower limits of RBC (3.3×10^12^/L for men versus 3.1×10^12^/L for women), HGB (10 g/dL for men versus 10.3 g/dL for women), HCT (28% for both sexes), WBC (1.9×10^9^/L for men versus 2.2×10^9^/L for women) and the NEU (0.5×10^9^/L for both sexes) compared to our study (Table 7). The complete blood count parameters according to sex showed a significant difference in our study between men and women when compared to other studies [23, 26]. For erythrocyte lineage, differences according to sex were observed in the values of RBC, HGB, HCT, MCV, MCH and MCHC, the highest values were observed for men. This difference was present according to age in all the studied provinces. These results are similar to previous studies [26-28] (Table 7).

Lower values of HGB corresponded to the lower limits in our population of women and in the Moroccan study done in the Casablanca region for both sexes with regard to the literature and to the Caucasian population could be explained by a higher frequency of the iron deficiency. On a national scale, indeed, the anemia affects more than a third of the Moroccan population with values observed in pregnant women (37.2%), sexually mature women (33%) and men (18%) [29]. So, hemoglobin diseases could be responsible because Morocco is one of the Mediterranean countries affected by thalassemia, classified 28th in the world. Prevalence of carriers of beta thalassemia is of the order of 3% [30]. Also, quality of food, standard of living as well as a higher index of gestation with, consequently, more gestational losses, represents factors that explain the above-mentioned differences. To reiterate, our study should be understood keeping in mind the impossibility of eliminating from our population of study the subjects presenting an iron deficiency or those affected by hemoglobin diseases. The French study of the Regional Institute for the Health (1989) confirmed the variation of the rate of the HGB according to sex [31]. Indeed, the values observed in a population of 77381 subjects from 6 to 65 years old were higher for men than for women with intervals, respectively, 13.1-17.2 g/dL and 11-15.4 g/dL. According to this study, this difference related to sex appears at puberty and persists in all age groups. This phenomenon could be explained, on one hand, by the difference in physiological changes and iron deficiencies in woman during pregnancy and menstruation, or, on the other hand, by androgenic erythropoiesis stimulation which is more prevalent in men [32]. In a Palestinian study, Sirdah et al showed a statistically significant difference of all the erythrocyte parameters between both sexes [33]. The same report references in studies regarding black African populations. Indeed, in the study of Lugada et al, which concerned 3000 Ugandan individuals, negative of HIV and a week from turning 92 years old, the values observed in this older population roughly 24 years older, were significantly higher for adult men than for adult women [27]: RBC 4.9×10^12^/L (3.8- 6.0×10^12^/L) for men versus 4.5×10^12^/L (3.7-5.3×10^12^/L) for women, HGB 14.1 g/dL (11.1-16.8 g/dL) for men versus 12.5 g/dL (10.1-14.3 g/dL) for women, HCT 40.7% (32.2-47.8%) for men versus 36.2% (29.6-41.4%) for women and MCV 84 fL (69.9-95.2 fL) for men versus 81.4 fL(67.7-92.6 fL) for women.

With regards to the constants of Wintrobe (MCV, MCH, MCHC), we noted that the literature admits comparable results for both sexes. However, their results diverge from ours which show differences between men and women. The minimal value of the MCV for men (77.4 fL) and for women (75.1 fL) is lower than that found in the French study (78.40 fL for men versus 80.20 fL for women) [16] and that published in the study of Rény Caquet (85 fL to both sexes) [19]. In our study, the median values of the global leukocyte numeration were significantly superior in woman than for men in the prefecture of Tangier-Assilah and the province of Chefchaouen. This report is true for all ages except for the age group 40-50 years when the median values were equal for both sexes (6.9 ×10^9^/L). Numerous studies made similar observations from different races populations [18, 20, 21, 33, 34]. This observation was contributed to specific physiological states (menstrual cycle or pregnancy) which slightly increase leukocytosis [22, 35]. On the contrary, certain authors found superior leukocytes values in men than in women of the same age [31, 36]. Other authors still discerned no significant difference between both sexes [37]. In the case of our study, men and women of the provinces of Tetouan and M'diq-Fnideq were found to have the same median value of leukocytes (7.0×10^9^/L). Moreover, the analysis of variation in categories of leukocytes according to the sex shows values of the median of NEU significantly higher for women than for men, is 3.9×10^9^/L versus 3.8 × 10^9^/L (p < 0.001). This variation was observed with regard to all age groups and provinces. The ethnic neutropenia, established well in numerous studies, does not seem to be connected to a sub-ethnic group as it was observed in black people of different geographical origins (Black Africa, America, etc) [25, 27]. The superior limit of the EOS was appreciably higher for men than for women of the same age except for the age group 30-39 years where the reference intervals of EOS were equivalent for both sexes (0-0.5 ×10^9^/L). Our results agree with those of the studies of Swaanenburg (40-400 × 10^6^/L for men versus 40-320 × 10^6^/L for women) and of Rakoto et al. (0.23 G/L for men versus 0.18 G/L for women) where higher values of EOS were observed for men [21, 34]. These results do not suit to those of El Graoui et al. (0.01-1.09 × 10^9^/L for women versus 0-1.0×10^9^/L for men) and Ben Amor et al. (0-0.35 ×10^9^/L for women versus 0-0.28 ×10^9^/L for men) where the higher values were found rather in women [18, 20].

The French study by the Center of Preventive medicine of Vandœuvre-lès-Nancy brings identical reference values of EOS for both sexes of adults [5]. Also, it was not reported in the literature of difference according to the sex of the numerations of BAS [34]. The reference intervals of BAS in our study were also similar between men and women (0-0.08×10^9^/L). Some studies of the international literature report lower rates of lymphocytes for women, this result was not found in the present study [34]. Indeed, we noted no significant difference between both sexes with regard to the numeration of lymphocytes within any of the age groups. Our results more closely align to those of the French study of the Regional Institute for the Health and the Tunisian study of Ben Amor [18, 31]. For the numeration of monocytes, we noted a significant difference between men and women. The results of the studies once compared to the rate of monocytes between both sexes were contradictory [38, 39]. In the study of Swaanenburg, the upper limits were superior in men (650 × 10^6^/L) than in women (580×10^6^/L) [21]. In the study of Rakato et al, the mean of the monocytes was superior in women (0.39 G/L) than in men (0.36 G/L) [34]. The published reference intervals of monocytes, in a study carried out on an adult Moroccan population of the Casablanca region (Morocco), were identical for both sexes (0.2 -1.1×10^9^/L) [20]. In our study, concerning the platelet lineage, the values of the median as well as the reference values of the platelet numeration were higher for women than men. These results are supported by numerous studies concerning the populations of different races [16, 18, 20, 23, 33]. This report could be attributed, on the one hand, to a hormonal influence and on the other hand, to higher iron losses in women due to menstruation and pregnancy [4]. This observation was found in all the established age groups and provinces. Additionally, the MPV found in our population was higher than that reported by some publications based on Caucasian populations (7.50-10.9 fL for women versus 7.4-10.8 fL for men) [16] and other African populations (8.5-12.7 fL for women versus 8.7-13.1 fL for men) [40].

## Conclusion

This study allowed us to establish the reference intervals of the complete blood count for a population of healthy adults represented by VBD of the Tangier-Tetouan region (Morocco). The differences found by this preliminary work in comparison to the literature data underline the necessity with to establish reference intervals of blood count appropriate to the Moroccan population through studies conducted within various regions of Morocco. These studies should specify the possible variations of the blood count for children, adolescents, the elderly and pregnant women, to avoid errors of diagnosis and allow clinicians to more specifically interpret the hematological examinations and to improve the quality of medical care offered to the patients.

### What is known about this topic

- The Complete blood count (CBC) is the most frequently requested laboratory test: it guides later additional investigations essential for diagnosing and monitoring the patient;
- The parameters of CBC are influenced by numerous factors such as sex, age, altitude, genetics, and diet. These factors vary according to geographic and ethnic origin;
- Laboratories are faced with the problem of providing reference values of hematological parameters specific to the Moroccan population and not having national recommendations.

### What this study adds

- Provide data of the hematology reference intervals in a healthy population of adults in the Northwest of Morocco;
- The reference values developed will be of immense benefit to most clinical trials requiring monitoring of hematological parameters;
- Need to establish reference intervals of CBC appropriate to the Moroccan population through studies conducted within various regions of Morocco.

## Competing interests

The authors declare no competing interests.
